# Transcriptome-wide Variability in Single Embryonic Development Cells

**DOI:** 10.1038/srep07137

**Published:** 2014-11-20

**Authors:** Vincent Piras, Masaru Tomita, Kumar Selvarajoo

**Affiliations:** 1 Institute for Advanced Biosciences, Keio University, 14-1 Baba-cho, 997-0035, Tsuruoka, Japan; 2 Systems Biology Program, Graduate School of Media and Governance, Keio University, 5322 Endo, 252-0882, Fujisawa, Japan

## Abstract

Molecular heterogeneity of individual molecules within single cells has been recently shown to be crucial for cell fate diversifications. However, on a global scale, the effect of molecular variability for embryonic developmental stages is largely underexplored. Here, to understand the origins of transcriptome-wide variability of oocytes to blastocysts in human and mouse, we examined RNA-Seq datasets. Evaluating Pearson correlation, Shannon entropy and noise patterns (*η*^2^ *vs.* *μ*), our investigations reveal a phase transition from low to saturating levels of diversity and variability of transcriptome-wide expressions through the development stages. To probe the observed behaviour further, we utilised a stochastic transcriptional model to simulate the global gene expressions pattern for each development stage. From the model, we concur that transcriptome-wide regulation initially begins from 2-cell stage, and becomes strikingly variable from 8-cell stage due to amplification and quantal transcriptional activity.

Numerous studies on single cells have shown that individual molecules (genes, proteins or metabolites), within an iso-genic and -phenotypic cell population, can display highly variable expression levels. For example, immunofluorescence flow cytometry showed that Sca-1 expressions in multipotent murine hematopoietic cells follow a Gaussian-like distribution^1^, and the monitoring of green fluorescent proteins in *Escherichia coli* displayed fluctuations in their expression levels over time^2^.

Such heterogeneous or noisy characteristics have shown to play pivotal roles for the survival of species to diverse environmental conditions or for cell fate decisions^3^,^4^,^5^. Notably, it was demonstrated that regulating stochastic noise in the levels of *comK* in *Bacillus subtilis* was necessary to control cell fate decision under nutrient-deficient conditions^6^. For *Caenorhabditis elegans*, the intestinal cell fate process from early embryonic lineage was shown to be regulated by the variability in *end-1* expression, providing the basis for incomplete penetrance^7^. These studies have identified crucial single molecules that regulate heterogeneity or variability of single cells within a population. However, little work has been performed to investigate global responses, comprising the entire spectrum of molecular species, within single cells. In particular, the extent of transcriptome-wide expressions noise in the early mammalian development has yet to be determined.

In this paper, to understand global gene expression structure and noise patterns of single cells during mammalian developmental stages, we investigated transcriptome-wide RNA-Seq expressions of several cells during human^8^ and mouse^9^ embryogenesis. A total of 7 human and 10 murine cell origins, from oocytes to blastocysts, were analysed using high-dimensional statistical techniques, such as correlation metrics^10^,^11^,^12^,^13^,^14^,^15^,^16^, Shannon entropy^17^,^18^,^19^ and noise analyses^20^.

## Results

### Single cell transcriptome expressions scatter increases along development stages

**Figure 1:**
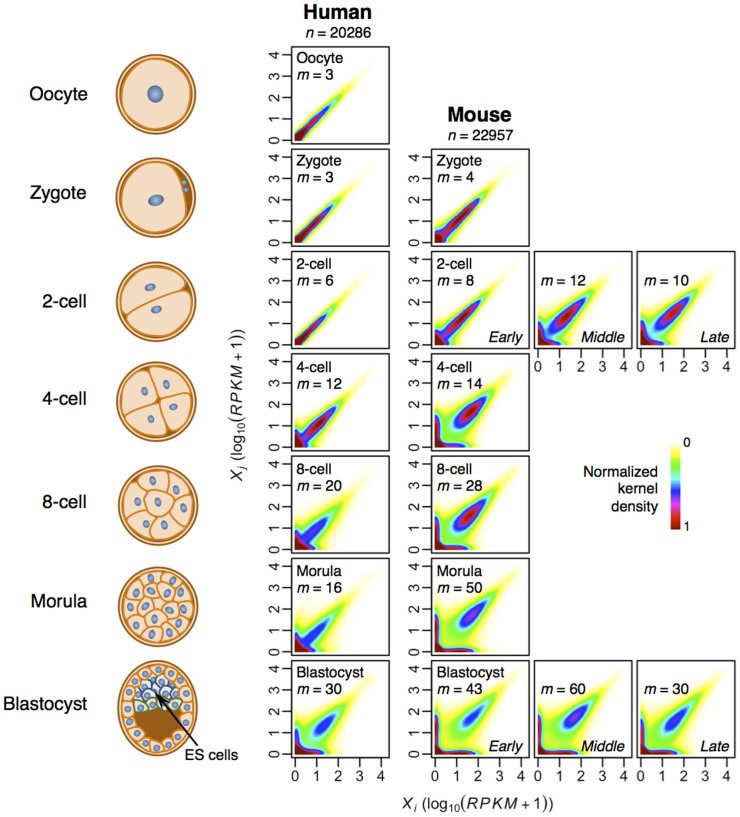
Gene expression structures for developmental stages. Gene expression distributions, estimated from kernel density estimation (*kde2d* R function) of all genes expressions (RPKM) between all possible pairs of single cells in human and mouse, from oocytes to blastocysts. *m* is the total number of single cells and *n* is the total number of genes.

To observe gene expression variability between 2 single cells at each developmental stage, we plotted pair-wise distributions of single cell transcriptomes (Figure 1). For human, we noticed global expressions scatter is tightly constrained up to 2-cell stage, after which the scatter widened, especially for lowly expressed genes. For mouse, the scatter widened noticeably from middle of 2-cell stage. These data suggest that transcriptome-wide expression distributions become more variable along the developmental stages.

**Figure 2:**
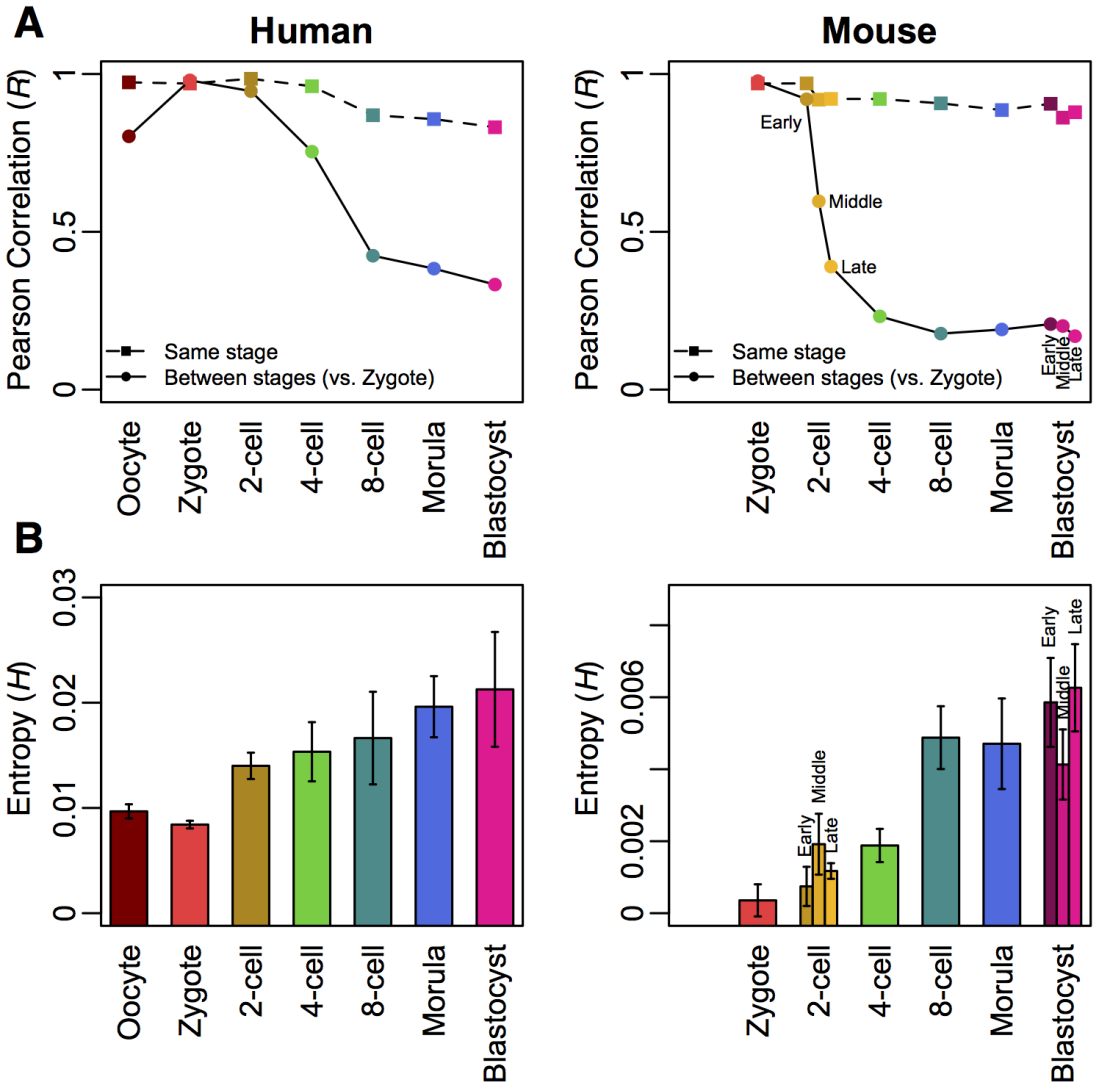
Phase transition in single cell transcriptomes. (A) Pearson correlation, *R*, between transcriptomes of cells of the same development stage (dotted lines) and or between transcriptomes of zygote and other stages (solid lines) for human and mouse. (B) Shannon entropy (*H*) of single cell transcriptomes (average for *m* cells, error bars indicate 1 s.d.).

To better understand the variability and the effects of technical and biological noises, we performed transcriptome-wide correlation (similarity) analysis^10^,^11^,^12^,^13^,^14^ by comparing the expressions of two cells from the same cell origin. Although large expressions scatters are observed, especially for late developmental stages (Figure 1), the global averaged Pearson correlation coefficients between single cells of the same stage, as expected^20^, are generally high (Figure 2A, dotted lines). However, the correlation coefficients between cells of distinct origins, are significantly lower (Figure 2A, solid lines and Supplementary Fig. S1 online). We further probed for non-linear relationships between the transcriptomes of cells of the same stage or different stages, and computed Spearman ranking metrics (non-linear monotonic relationships), distance correlation^15^ (strict statistical dependence) and maximum information coefficient^16^ (linear and non-linear associations) (see Materials and Methods). Remarkably, all metrics revealed similar trends compared with Pearson correlations (Supplementary Fig. S2 online). These results indicate that the global transcriptional program of developmental cells clearly deviates along the stages in time, with faster rate of deviation occurring for mouse when compared with human (Figure 2A, solid lines).

Next, we assessed the diversity of single cell transcriptomes using Shannon entropy, which measures the disorder of a high-dimensional system, where higher values indicate increasing disconnection between variables and zero value indicates order^17^,^18^,^19^ (Materials and Methods). For both human and mouse, Shannon entropies remained low in early stages but gradually increased from 2-cell (human) or 4-cell (mouse) stage, to reach high values for morula and blastocyst (Figure 2B). This result, therefore, shows the disconnection or diversity of transcriptome-wide expressions increases during mammalian development.

### Transcriptome-wide noise increases during development stages

**Figure 3:**
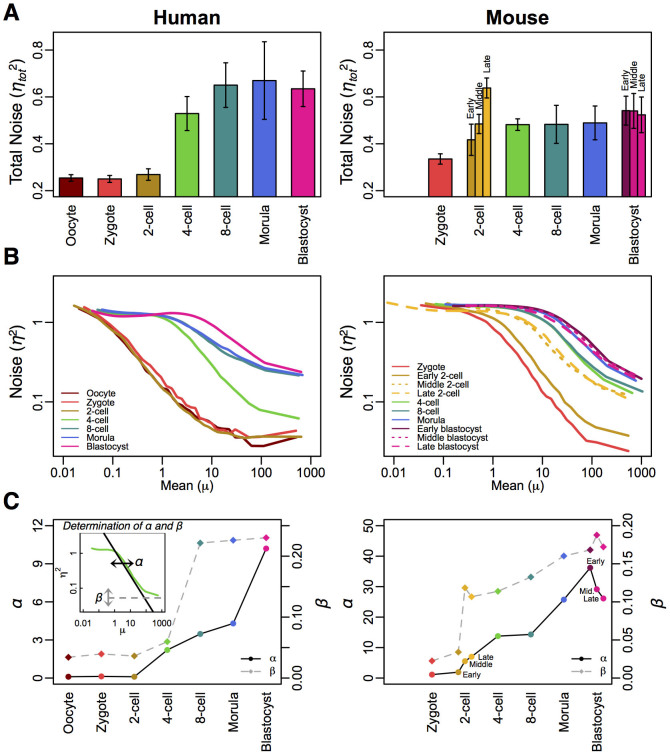
Transcriptome-wide noise patterns. (A) Total noise (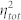) of single cells for each development stage (average for *m* cells, error bars indicate 1 s.d.). (B) Noise (*η*^2^) *vs.* mean (*μ*) expression patterns for each development stage. (C) Plots of *α* and *β* against cell stage. The insert illustrates how *α* and *β* are determined.

To further understand the effects of increasing entropy and diversity in single cell transcriptomes during embryogenesis, we quantified single cells' expressions scatter by computing transcriptome-wide average noise (renamed as total noise), 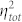, i.e. summing the squared coefficient of variation^21^, defined as the variance (*σ*^2^) of expression divided by the square mean expression (*μ*^2^), for all genes (*i*) between all possible pairs of single cells (Materials and Methods). We observed that 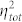 is low during initial embryonic cell differentiation, but increases at later stages with significant increase from 2- to 4-cell stage onwards (Figure 3A). We also compared total noise for embryonic stem, normal somatic and cancer cells, and found similar values as obtained for later stage development cells (Supplementary Fig. S3 online). These data indicate that total noise stabilises at ~0.7 and may not increase further.

So far, we have analysed the entire transcriptome without setting any expression cut-off. It is known that lowly expressed genes in single cells are dominated by stochastic and/or technical noises, which reduce their between cells correlation values, while highly expressed genes show more deterministic expressions^22^. Thus, we delineated total noise to investigate average noise for every group of 500 genes, between pairs of single cells, as a function of mean expressions (*μ*) starting from the lowest expressions.

As expected, we noticed noise is relatively high for the lowly expressed portion of the transcriptome (*η*^2^(*μ*) ~ 2 for *μ* < 0.1 for all cell types) (Figure 3B). This portion of transcriptome is usually discarded due to low signal-to-noise ratios. Considering noise patterns above this threshold (*μ* ≥ 0.1), we observed all patterns followed the relationship, *η*^2^(*μ*) = *α*/*μ* + *β*, where *α* and *β* are proportionality constant and asymptotic value, respectively (Supplementary Fig. S4 online). That is, noise scales with the inverse of mean values reaching asymptotic values at higher expressions for all cell types and species, including other embryonic stem cells, somatic cells and human cancer cells^23^ (Supplementary Fig. S3 online). Notably, this relationship was also previously observed for other high-throughput datasets^21^,^24^.

Fitting the noise pattern of each cell type and species, we observed *α* and *β* increase during the human development process; oocyte, zygote and 2-cells stages show similar patterns, 4-cell stage shows transitional values, while 8-cell, morula and blastocyst stages reach higher values. Similar trends were also found for mouse, nevertheless, the transition between low and high values occurred earlier at middle of 2-cell stage (Figure 3C). These results show that transcriptome-wide noise increased along the developing stages and stabilises from 8-cell stage. Note that other grouping sizes, e.g. 50, 100, 1000 genes (Supplementary Fig. S5 online), did not affect the overall increase in transcriptome-wide noise along the developing stages.

### Deciphering the origins of noise patterns using a stochastic transcriptional model

**Figure 4:**
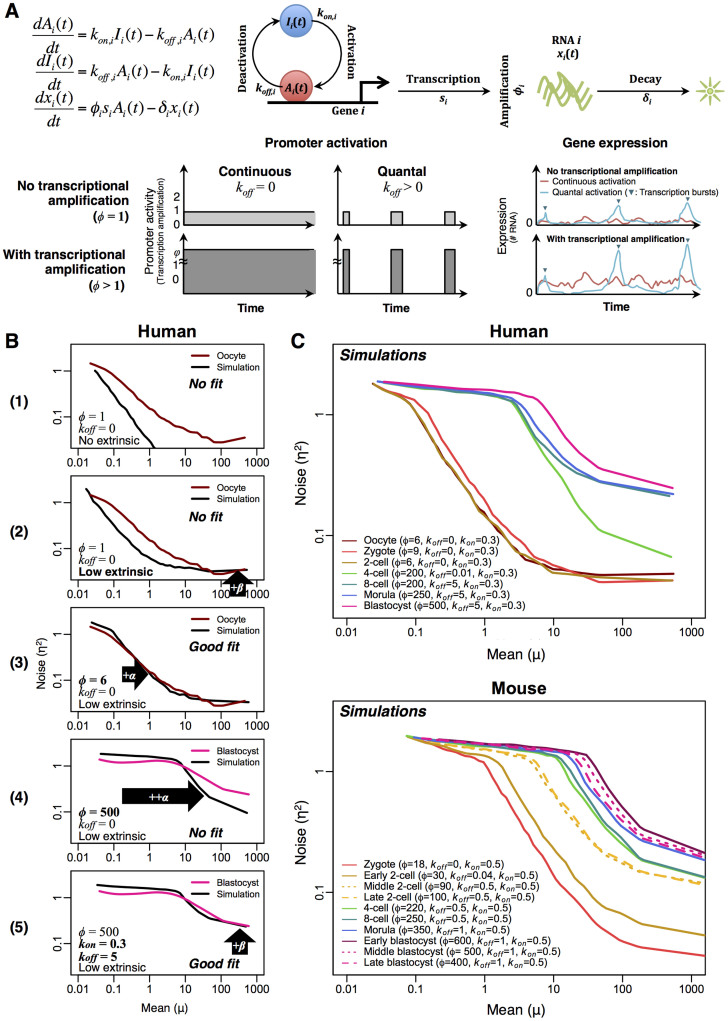
Simulations of transcriptome-wide expressions. (A) Top: single cell transcriptional model. Bottom: promoter activity (left and middle panels) and gene expressions (right panels) for continuous and quantal simulations, without and with transcriptional amplification. (B) Simulations (panel 1, black curves) using continuous activation, no amplification and no extrinsic noise. Addition of extrinsic noise only (panel 2), and with low transcriptional amplification (panel 3) allowed better fit of simulations to early development stages patterns (e.g. oocyte, brown curve). Higher amplification with extrinsic noise (panel 4). Higher amplification and quantal activation with extrinsic noise (panel 5). Panel 5 shows a good fit with late stage developmental cells. (C) Simulated noise *vs.* mean expression patterns for all developmental stages in human (top) and mouse (bottom). Note that *k_on_* = 0.5 was used for mouse simulations and *k_on_* = 0.3 for human.

To understand the transcriptional mechanisms governing increasing noise patterns along the development process, we utilised a single cell transcriptional model based on Gillespie stochastic algorithm. We simulated transcriptome data constituting 20,000 gene ‘units', where each gene dynamics is governed by 3 ordinary differential equations with 5 kinetic parameters^25^,^26^; transcription rate (*s*), degradation rate (*δ*), promoter activation (*k_on_*) and deactivation (*k_off_*) rate constants, where both continuous gene promoter activation (*k_off_* = 0) and quantal (bursty) dynamics (*k_off_* > 0) can be simulated (Figure 4A). The transcriptional amplification process^27^, i.e. number of transcripts produced per activation event, is controlled by *φ*.

Using the model, we simulated gene expressions for various conditions by controlling the 5 kinetic parameters. Firstly, simulations were performed by choosing the transcription (*s*) and degradation (*δ*) rate parameters for each gene from statistical distributions found in experimental data^28^,^29^ (Supplementary Fig. S6 online). The three other parameters were kept at default constant values (*φ* = 1, *k_off_* = 0 and *k_on_* = 0.5). In this theoretical setting, generated noise is entirely intrinsic (and Poisson), and decreased with mean expressions with *α* constant and *β* = 0 for the whole range of gene expressions (Figure 4B, panel 1). It is conceivable that none of the development stages follow this idealised condition, which does not contain noise due to extrinsic or other non-Poisson factors, such as technical noise^30^,^31^.

To consider expression-independent noise in our simulations, such as technical and environmental variability, we included additive and multiplicative random white noise^32^,^33^ to our simulated expressions. As a result, we observed an increase of *β*, while *α* remained unchanged (Figure 4B, panel 2). This is because the white noises do not interfere with the transcriptional regulation.

Although none of the development stages fitted this pattern, we observed the expression-independent noise values at higher expression levels of early stages could be achieved by setting a certain threshold of random white noise, such as *β* = 0.03 for human and 0.02 for mouse (Supplementary Fig. S4 online). We, therefore, set these values as the level of extrinsic noise for all the subsequent simulations, assuming it remains unchanged across each cell type for a particular species.

Next, we increased the number of transcripts produced per activation event, which resulted in amplification of the scatter in expressions (Supplementary Fig. S7 online), with corresponding increase in noise structure (Figure 4B, panel 3). Since *φ* controls transcriptional amplification, it is conceivable that increasing *φ* will proportionally increase *α*. However, *β* was not affected.

For human, the simulated noise structures matched experimental patterns of oocyte, zygote, and 2-cell stages for low *φ* and 4-cell stages for high *φ* (Figure 4C). For later stages, although higher values of *φ* improved the simulation results, as noted earlier, *β* could not be increased to fit the experimental patterns (Figure 4B, panel 4). For mouse, the trend of increasing over developmental stages is also observed (Figure 3B), however, the values are generally higher for early stages (Figure 4C and Supplementary Fig. S8 online). Like human data, the mouse simulations also did not match for the values of *β* (Supplementary Fig. S8 online).

To improve the simulation results, that is to specifically increase *β* values, we next explored the parameters governing bursty transcriptional dynamics: *k_on_* and *k_off_*. Previous experiments in mouse ES cells^34^ suggest 0.1 ≤ *k_on_* ≤ 2.5 h^−1^ (median, 0.5 h^−1^) and 3 ≤ *k_off_* ≤ 200 h^−1^ (median, 14 h^−1^). To obtain *β* values that fit the human experimental patterns of later development stages, we initially set *k_on_* = 0.5 and increased *k_off_* to 5. However, the value for *β* obtained from simulations was still too low, and further increase of *k_off_* did not produce major increase in *β*. We, therefore, reduced the value of *k_on_* such that *k_on_* = 0.3 to obtain a good fit (Figure 4B, panel 5). For mouse, *k_on_* = 0.5 and *k_off_* > 0.5 were required to fit the experimental *β* values. Again, further increases of *k_off_* did not significantly increase *β* (Supplementary Fig. S8 online).

Overall, from these simulations, we concur that distinct transcriptional mechanisms govern transcriptome-wide expressions during embryonic development. Notably, we showed that *α* is governed solely by transcriptional amplification whereas *β* is controlled by both extrinsic noise and quantal transcriptional activation.

## Discussion

Recent studies on single cells have shown that individual molecules (genes, proteins or metabolites), within a homogenous cell population, can display highly variable expression values^1^,^2^,^6^,^7^,^35^,^36^. This variability has been linked to biological noise or the stochastic nature of molecular network regulations^37^,^38^,^39^. However, there is a general lack in the investigation of global regulatory mechanisms at an omics-wide scale for single cell behaviour. Studying global properties has been instrumental in interpreting collective mechanisms of living organisms, for example, the innate immune response to invading pathogens^13^ or the attractor states of cell differentiation process^40^. Here, to understand the global noise patterns of single developmental cells, we investigated RNA-seq transcriptome-wide expressions of oocytes to blastocysts in human and mouse.

Firstly, by studying the distribution of gene expressions between single cells, we observed the expression scatter increased from 2-cell to 4-cell stage onwards in both human and mouse (Figure 1). Next, we examined the Pearson correlation and Shannon entropy for each developmental stage. Again, we observed that expressions become more variable from the 2-cell stage (Figure 2). Subsequently, the global noise character of single cells was investigated by quantifying the squared coefficient of expression variations over mean expression values. Here, we observed clear transition of noise patterns occurring between 2-cell and 8-cell stage (Figure 3).

To understand the noise patterns, we developed a stochastic transcriptional model and estimated the parameter values to match each developmental cell pattern (Figure 4). From the model, we concur that the early developmental stages are mainly dominated by low transcriptional activity. For these stages, the number of transcripts produced per activation event, *φ*, is low. The lower overall transcription in oocytes and early zygote is consistent with i) transcriptional silencing and ii) stochastic degradation of maternal RNA that has been observed from oocytes to 4-cell stage in humans^41^,^42^. Transcriptional silencing is likely due to chromatin condensation state that prevents transcriptional machinery from reaching gene promoters^41^,^43^,^44^.

**Figure 5:**
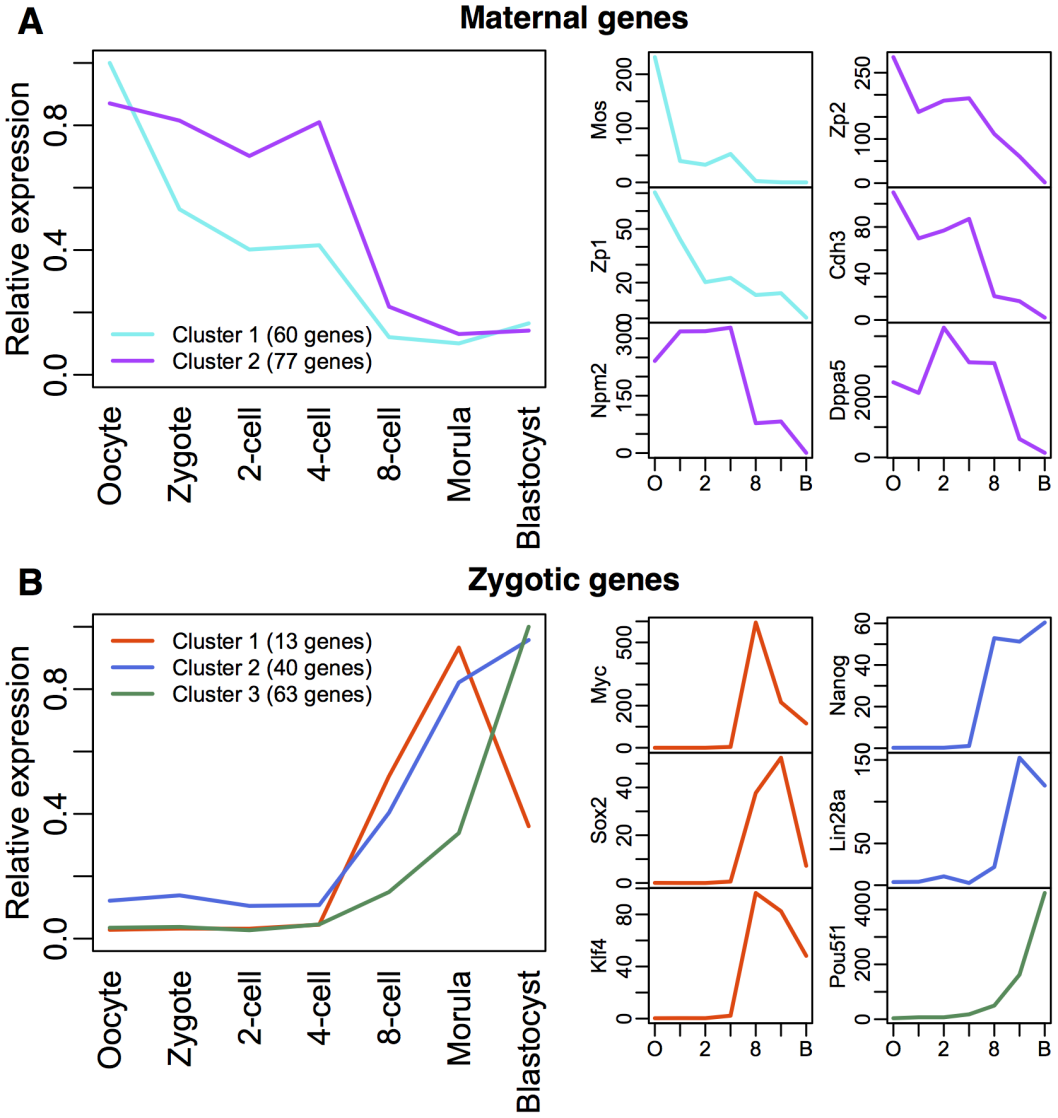
Gene expression profiles of maternal and zygotic genes during development. Average gene expression profiles of (A) maternal (2 clusters) and (B) zygotic (3 clusters) genes during human embryo development. Clusters were obtained using k-means clustering. From the initial lists of genes obtained from Xue et al.^45^ (Table S1), we retained maternal genes with expression values reaching peak before 4-cell stages and zygotic genes with expression values reaching peak from 8-cell stage onwards. Right panels show the expression values in all development stages of individual maternal genes (*Cdh3, Dppa5, Mos*, *Npm2, Zp1* and *Zp2*; obtained from Kocabas et al.^46^) expressed in oocytes, and zygotic genes involved in later embryonic stages and stem cells (*Myc, Klf4*, *Pou5f1 (Oct4), Sox2, Lin28a* and *Nanog*; obtained from Takahashi et al.^47^ and Yu et al.^48^).

To track the gene expression profiles of common maternal^45^,^46^ and zygotic genes^45^, we plotted their relative expressions (Supplementary Fig. S9 online), and found 2 and 3 major temporal clusters, respectively, for 137 maternal and 116 zygotic highly expressed genes (Figure 5 and Table S1 online). Notably, the maternal genes (e.g. *Cdh3, Dppa5, Mos*, *Npm2, Zp1*, *Zp2*) showed dominant decay profiles of RNA expressions, indicating lack of transcription process (Figure 5A). However, the zygotic genes and genes expressed in embryonic stem cells^47^,^48^ (e.g. *Klf4*, *Lin28a, Myc*, *Nanog, Pou5f1, Sox2*) showed transcriptional process significantly increasing after the 4-cell stage (Figure 5B). The high transcriptions can be due to instructive signaling pathways, or multiple rounds of transcription reinitiation by RNA polymerase^26^,^49^,^50^. The observation of high transcriptome-wide noise for the middle stage developmental cells indicates the generation of heterogeneity in gene expressions between individual cells. Such heterogeneity has been shown to be necessary for cell fate diversifications^1^,^6^.

For the later stage developmental cells, on top of high transcriptional process, the cells possess quantal activation of most transcription factors, or are subject to more extrinsic variability such as phenotypic diversity among individual cells. These factors increase the general expression scatter and noise levels. However, investigating expression-independent random noise in our single cell transcriptional model simulations suggest that the levels of extrinsic and/or technical noise in our RNA-Seq data for all cells are relatively low (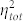 ~ 0.25). That is, the relatively high levels of noise for later stages stem from quantal activation rather than technical biases, or in certain cases, such as blastocyst cells, may result from phenotypic variability, as blastocysts consist of different cellular subtypes. Conversely, since phenotypic variability among more homogenous 8-cell stage is similar to blastocyst (Figure 1), we believe that quantal promoter activation is crucial for the increase of noise scatter along development stages. Notably, such quantal promoter activation has been noted to occur for single cell organisms such as *E. coli*^3^, and has been shown to be important for the cell fate decision of *B. subtilis*^6^.

Overall, our investigations on the transcriptome-wide expressions of the early mammalian developmental stages reveal increasing variability and noise patterns across the mammalian development process. These data suggest different stages of the cell differentiation process can be better understood by investigating the transcriptome-wide noise patterns. In conclusion, our systemic approach provides novel insights into the transcriptome-wide expression and noise patterns for development cells, and the underlying nature of the transcriptional mechanisms.

## Methods

### Single cell datasets

Single cell RNA-Seq datasets were downloaded from Gene Expression Omnibus (GEO) database from previously published data for 7 human^8^ (GSE36552) and 10 mouse^9^ (GSE45719) developmental stages. All datasets were obtained through Illumina sequencing systems. Each dataset contains the RPKM values (Reads Per Kilobase Mapped) for *n* ~ 20,000 genomic features, which is proportional to the number of transcripts of coding and non-coding genes (and splicing variants).

### Correlation analyses

To quantify transcriptome variability we utilised correlation metrics, which are widely employed to compare global relationships between high-throughput datasets^10^,^11^,^12^,^13^,^14^.

The Pearson correlation between two transcriptomes, ***X*** and ***Y***, containing *n* gene expressions, is obtained by 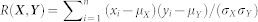, where *x_i_* and *y_i_* are the *i*^th^ observation in the vectors ***X*** and ***Y*** respectively, *μ_X_* and *μ_Y_*, the average values of each transcriptome, and *σ_X_* and *σ_Y_*, the corresponding standard deviations. The Spearman correlation coefficient between transcriptomes ***X*** and ***Y*** is defined by 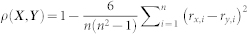, where *r_x,i_* and *r_y,i_* are the ranks of the *i*^th^ observations *x_i_* and *y_i_*, in vectors ***X*** and ***Y*** respectively. Both correlations were computed using the *cor* function of R *stats* package (http://www.r-project.org/).

Pearson and Spearman correlations respectively measure linear and non-linear monotonic relationships between two vectors, where *R* = 1 (respectively *ρ* = 1) if the two vectors are identical, and *R* = 0 (respectively *ρ* = 0) if there is no linear or monotonic relationships between the vectors. However, both metrics do not detect other non-linear relationships, therefore null correlation values do no imply statistical independence. To obtain a more stringent measure of statistical dependence between transcriptomes ***X*** and ***Y***, we used Distance Correlation^15^ (*dCor*), where *dCor*(***X***, ***Y***) = 0 if and only if the two vectors are statistically independent. Maximum Information Coefficient^16^ (*MIC*) can also be used to detect other types of non-linear associations between the transcriptomes, by calculating mutual information of the vectors using an automated non-parametric approach for binning. The computation of Distance Correlation values was performed using the *dcor* function of the R *energy* package, and the computation of Maximum Information Coefficient with the *mine* function of the R *minerva* package, with default parameters.

### Entropy analysis

To assess the diversity of single cell transcriptomes, we used Shannon entropy^17^,^18^,^19^. Shannon entropy measures the disorder of a high-dimensional system, where higher values indicate increasing disorder. Entropy of each single cell transcriptome, ***X***, is defined as 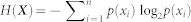, where *p*(*x_i_*) represents the probability of gene expression value *x* = *x_i_*. Entropy values were obtained through binning approach and the number of bins, *b* = 26, was determined from the data using Doane's rule^51^, such as 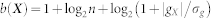, where *g_X_* is the skewness of the expression distribution of each sample, and 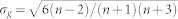. The computation of entropy values was performed using the maximum likelihood implementation (*entropy.empirical*) of the R *entropy* package.

### Transcriptome-wide average noise

To quantify between single cells' expressions scatter, we computed Transcriptome-wide average noise for each cell type, defined as 
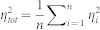
, where *n* is the number of genes and 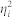 is the pairwise noise of the *i*^th^ gene (variability between any two cells), defined as 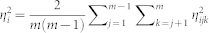, where *m* is the number of cells and 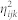 is the expression noise of the *i*^th^ gene, defined by the variance divided by the squared mean expression^18^ in the pair of cells (*j*,*k*), such as 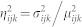, where *μ_ijk_* = (*x_ij_* + *x_ik_*)/2 is the average value of the *i*^th^ gene in the pair of single cells (*j*,*k*), and 
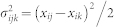
, is the corresponding variance.

### Transcriptome-wide noise patterns

To elucidate transcriptome-wide noise patterns, we sorted the transcriptome into groups of *w* = 500 genes from low to high expression values for each pair of cells (*j*,*k*). We formed *G* = *n*/*w* groups, and obtained the average gene expression of each group for each pair of cells, 
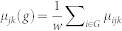
, and the average gene expression noise of all genes contained in the group, 
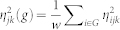
, for each pair of cells. We finally obtained characteristic whole transcriptome noise patterns 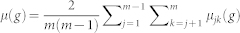 and 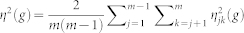 by averaging the patterns of all pairs of cells.

For simplicity, we used *μ* = *μ*(*g*) and *η*^2^ = *η*^2^(*g*) in the main and following texts. As a result, the reported curve for each stage is the average pattern of all single cells pairs. We fitted noise as function of mean expressions, *η*^2^ = *f*(*μ*), 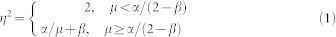
using nonlinear least squares piecewise curve fitting with the *nls* R function, to obtain the values of *α* and *β* for each pair of cells in all development stages.

### Transcriptome simulations

Each transcriptome model consists of a set of 20,000 genes, and each gene's expression is obtained from the stochastic simulation^52^ of a telegraph process^25^, such as the system of rate equations that governs gene expression *x_i_* of the *i*^th^ gene is 
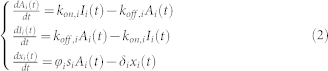
where the promoter activation of each gene is defined by a 2-state model, active (*A_i_*) and inactive (*I_i_*) states, and the transition rates between states are defined by two parameters, *k_on,i_* and *k_off,i_*. *s_i_* is the transcription rate when the promoter is active, *φ_i_* is the transcription amplification factor and *δ_i_* is the degradation rate constant. The distribution of RNA degradation rate constants, *δ_i_*, was obtained from transcriptome-wide RNA half-lives data in differentiating mouse ES cells^28^ and human B cells^29^, and fitted to a lognormal distribution with mean (log scale) and standard deviation parameters, *μ* = −2.24 and *σ* = 0.61 for mouse and *μ* = −1.58 and *σ* = 0.73 for human (Supplementary Fig. S6 online).

RNA transcription rates, *s_i_*, were estimated from the same data^28^,^29^, such as *s_i_* = *x_i_δ_i_* (*k_on,i_* + *k_off,i_*)/(*φ_i_k_on,i_*), where *x_i_* is the gene expression value (read counts) of the *i*^th^ gene, taken from a Zipf's law distribution with exponent *r* = 0.8 to fit the expression range in our data (Supplementary Fig. S6 online, inserts). As a result, we fitted the estimated values using a lognormal distribution for *x_i_δ_i_*, with parameters *μ* = 0.17 and *σ* = 2.67 for mouse and *μ* = 0.73 and *σ* = 2.53 for human (Supplementary Fig. S6 online). Since the model simulates integer read counts expressions, to obtain corresponding RPKM values, we multiplied our simulated values by a normalization constant, *Γ*, defined as the average ratio between RPKM values of all genes and corresponding number of reads (*Γ* = 0.03 for the human dataset, and 0.12 for mouse).

To generate transcriptome-wide expressions, we assigned different values for degradation rate constant, *δ_i_*, and transcription rate, *s_i_*, for each gene, and set the values of *φ_i_*, *k_on,i_* and *k_off,i_*, identical for all genes. To test variable values of *φ_i_*, *k_on,i_* and *k_off,i_* for each gene, we generated transcriptome-wide expressions using a Poisson distribution for *φ_i_* with parameter *λ* = *φ_i_*. Our result showed no noticeable difference in the patterns between fixed value or Poisson distributed *φ_i_*.

Similarly we simulated transcriptomes with promoter activation kinetics that vary between genes. We estimated the distributions of *k_on,i_* and *k_off,i_* from previously observed experimental distributions of promoter ‘on' and ‘off' time intervals^34^ (*τ_on,i_* and *τ_off,i_*). From the data, we observed the distributions of *τ_on,i_* and *τ_off,i_* could be approximated by an exponential distribution with parameter *λ* = 10, and a lognormal distribution with parameters *μ* = 0.69 and *σ* = 1 respectively. Since *k_on,i_* = 1/*τ_off,i_* and *k_off,i_* = 1/*τ_on,i_*^53^, we found median *k_on,i_* = 0.5 h^−1^ (0.1 ~ 2.5 h^−1^ range) and median *k_off,i_* = 14 h^−1^ (3 ~ 200 h^−1^). We then compared the simulations using fixed *k_on,i_* = 0.5 and *k_off,i_* = 14 or variable *k_on,i_* and *k_off,i_* and found no significant change in the patterns.

To account for non-intrinsic variations, we introduced different levels of additive and multiplicative white (Gaussian) noise to the simulated data^32^,^33^. Additive noise is achieved by adding a different random value to each gene in each cell, and multiplicative noise, by multiplying all gene expressions in the same cell by a random number such as, 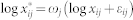, where 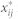 is the expression of *i*^th^ gene in the *j*^th^ cell including non-intrinsic noise, *ε_ij_* represents additive noise for the and *i*^th^ gene in the *j*^th^ cell, and *ω_j_* is the multiplicative noise that affects the *j*^th^ cell. *ε_ij_* is chosen from a normal distribution (*ε_ij_* ∈ *N*(0,*ε* log*x_ij_*)) with mean and standard deviation parameters, *μ* = 0 and *σ* = *ε* log*x_ij_*, where *ε* represents the level of additive noise. *ω_j_* is log-normal distributed, such as *ω_i_* ∈ *N*(0,log*ω*) with parameters *μ* = 0 and *σ* = *ω* respectively, and *ω* is the level of multiplicative noise. We used *ε* = 0.08 and *ω* = 0.1 to simulate non-intrinsic noise of human dataset (equivalent to *β* ~ 0.03), and *ε* = 0.06 and *ω* = 0.1 for mouse (*β* ~ 0.02).

## Author Contributions

V.P. and K.S. analysed and interpreted the data, developed the gene expression model, and performed the simulations. V.P., M.T. and K.S. wrote and reviewed the manuscript.

## Supplementary Material

Supplementary Information Figure S1-S9 and Table S1
