# Supplementary material for: Transcriptome-wide Variability in Single Embryonic Development Cells
Source: Sci Rep. 2014 Nov 20;4:7137. doi: 10.1038/srep07137 (PMC4238013; doi:10.1038/srep07137)
Supplement: Supplementary Information — Figure S1-S9 and Table S1 [file srep07137-s1.pdf]

Transcriptome-wide Variability in Single Embryonic Development Cells

Vincent Piras, Masaru Tomita and Kumar Selvarajoo*

Supplementary Information

Figure S1. Pearson correlations of single cell transcriptomes. Pearson correlations, *R,* of single cell transcriptomes at same (diagonal) and different developmental stages.

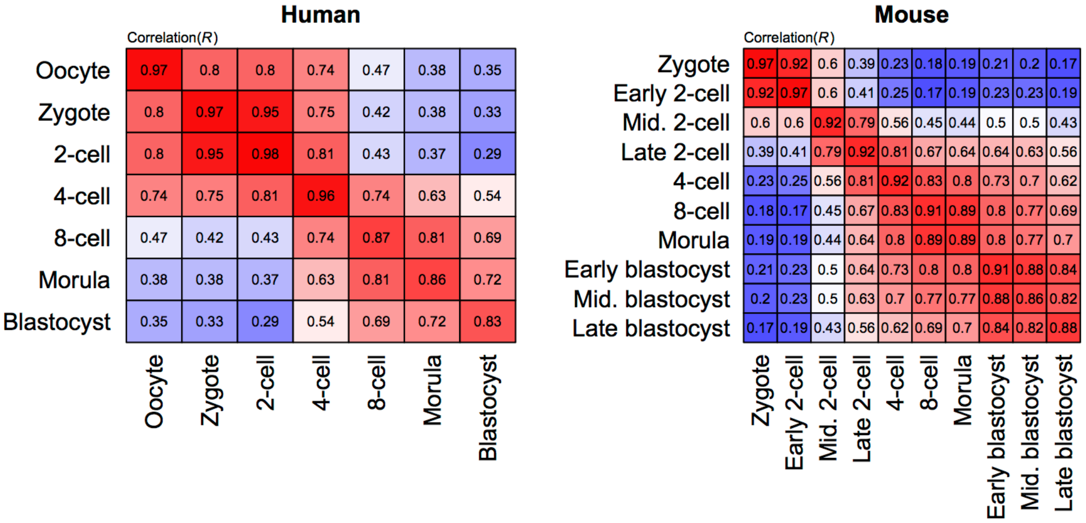

Figure S2. Non-linear correlations of single cell transcriptomes for human and mouse development stages. Spearman correlation, ρ **(A)**, Distance Correlation, *dCor* **(B)**, and Maximum Information Coefficient, *MIC* **(C)** between transcriptomes of cells of the same development stage (dotted lines) and or between transcriptomes of zygote and other stages (solid lines) for human and mouse.

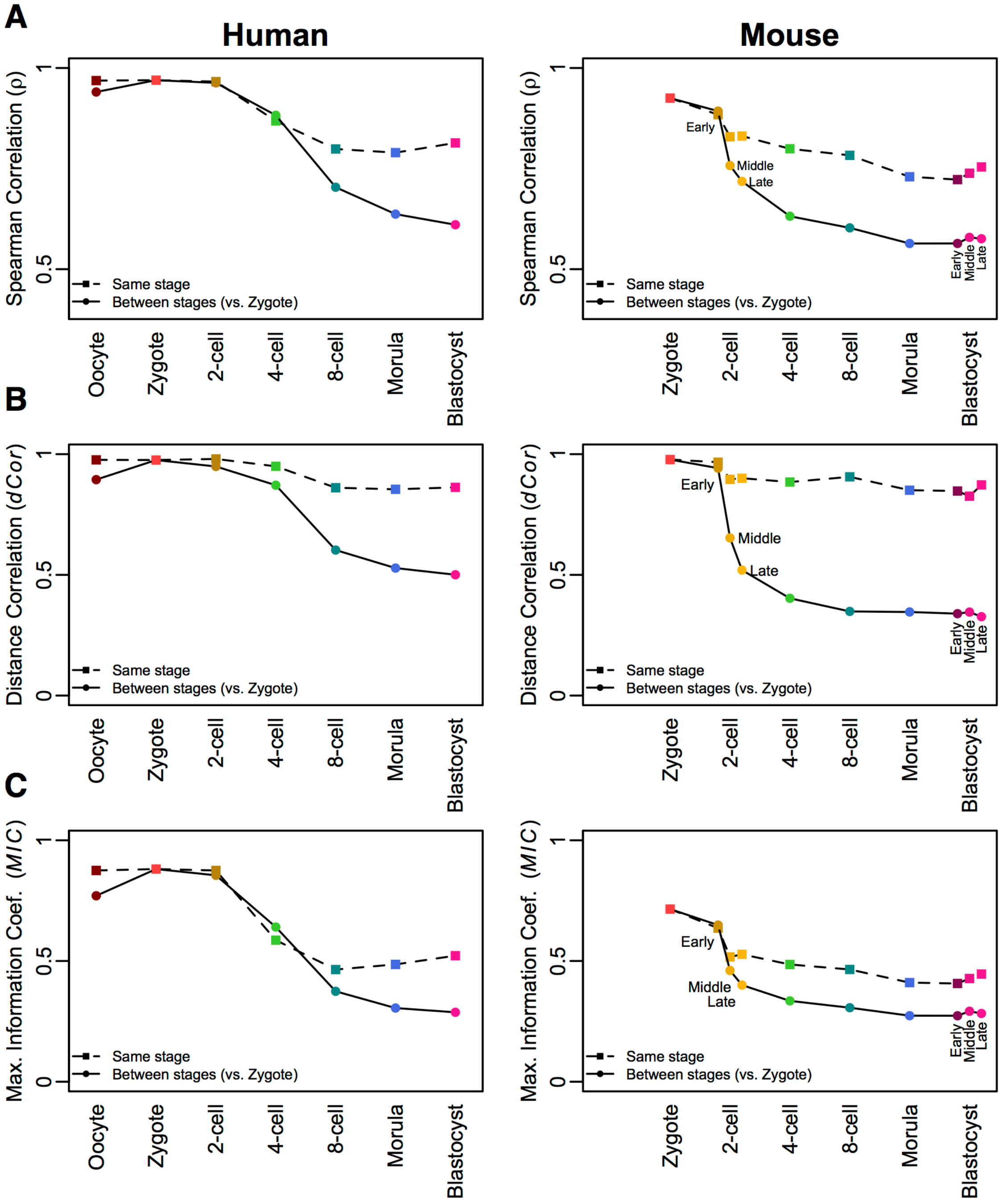

Figure S3. Total transcriptome noise in single somatic, cancer and ES cells. (A) Average total transcriptome noise (η^2_{tot}) of single ES, somatic (normal melanocyte), and cancer (circulating melanoma, SKMEL5 and UACC257 melanoma, LNCaP and PC3 prostate cancer, T24 bladder cancer) cells stage (average for *m* cells, error bars indicate 1 s.d.). Data was obtained from GEO dataset GSE38495 (Ramsköld et al., *Nat. Biotechnol.* **30**, 777–782 (2012)), and contains expressions (RPKM values) of $n = 21517$ genes. **(B)** Noise (η^2) vs. mean (μ) expression patterns for each cell type.

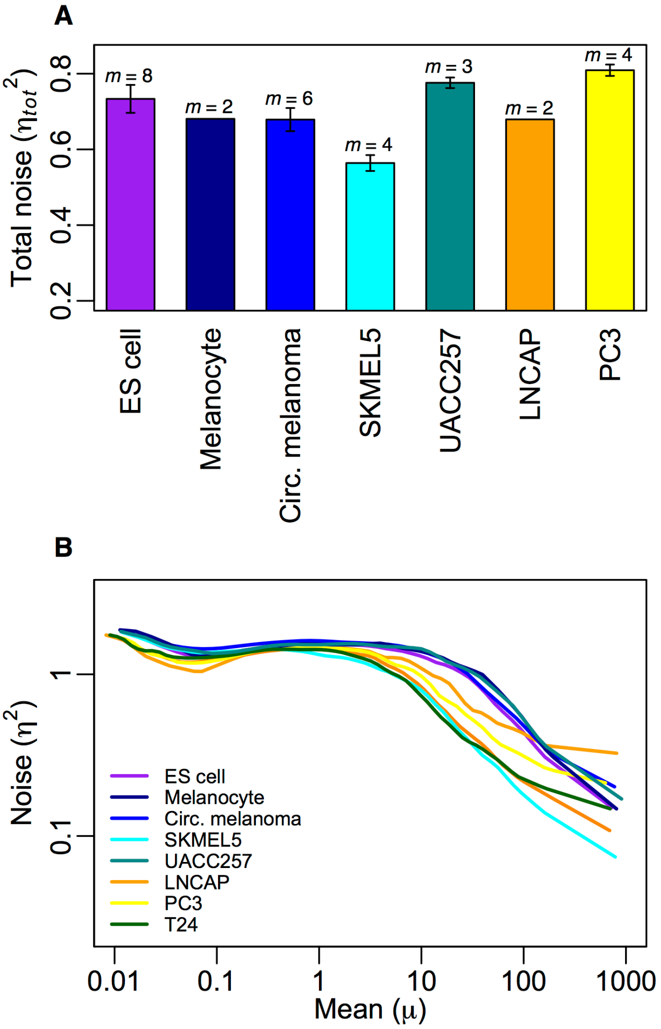

Figure S4. Fitting of the transcriptome-wide noise patterns. Noise (η^2) vs. mean (μ) expression patterns follow the relationship $\eta^2(\mu) = \alpha/\mu + \beta$ for each human **(A)** and mouse **(B)** development stage. Values of α and β were determined by non-linear squares fitting.

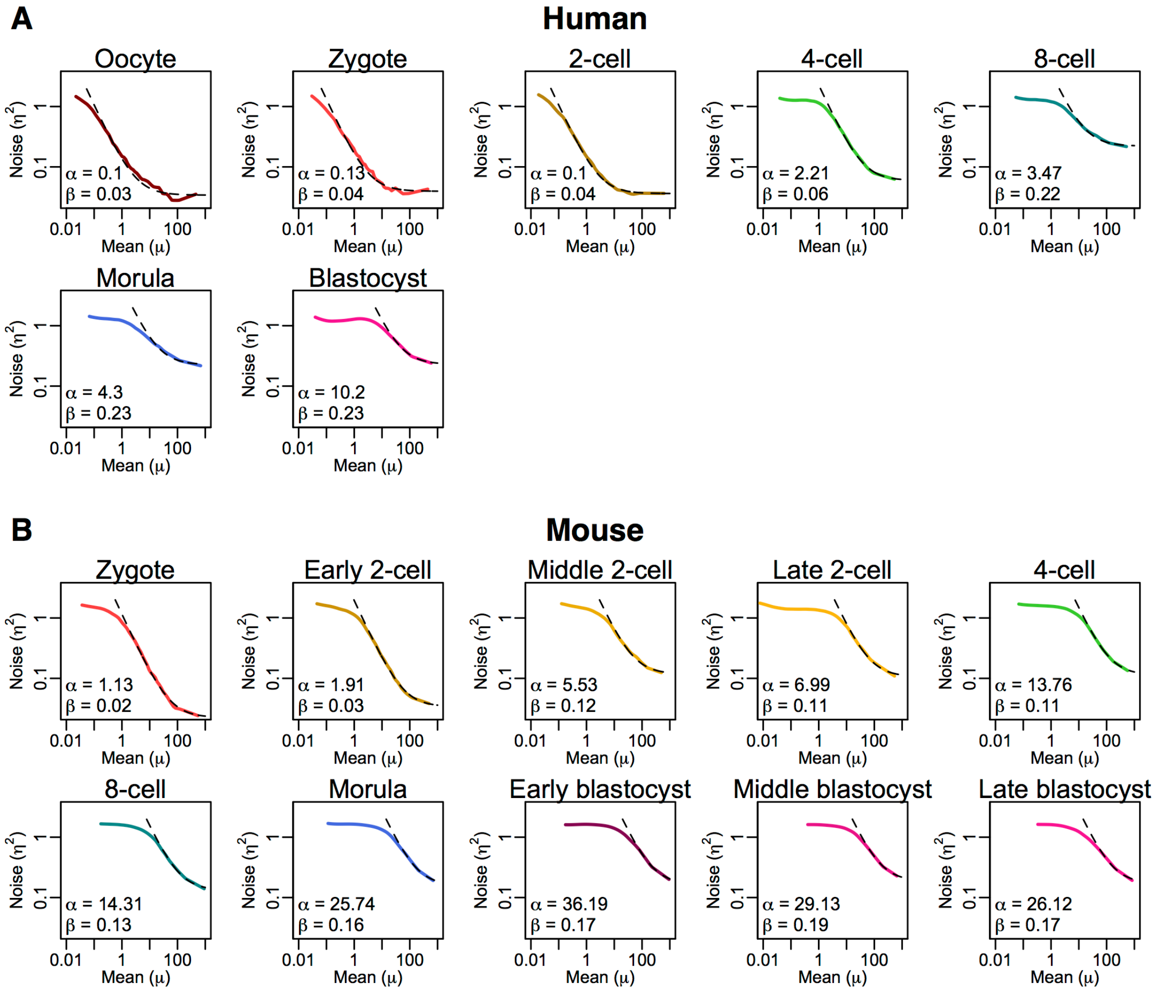

Figure S5. Transcriptome-wide noise patterns for each development stage when varying grouping size. Transcriptome-wide noise patterns were obtained by sorting the transcriptome into groups of $w = 50$, 100, 500 or 1000 genes from low to high expressions, averaging the mean expression (μ) and noise (η^2) of all genes in each group for all pair of cells. We chose $w = 500$ for analysis in the maintext.

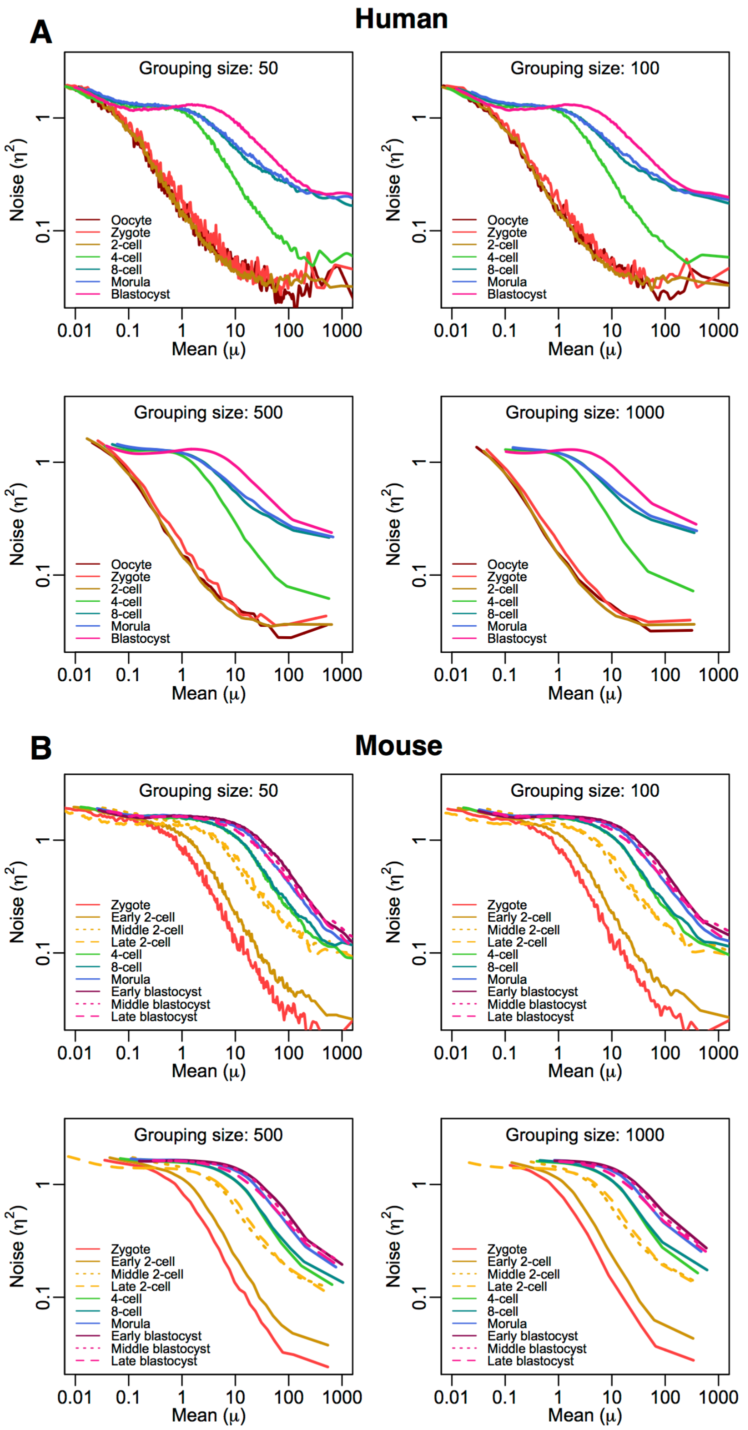

Figure S6. Distributions of RNA degradation and transcription rates constants. (**A**) The distribution of degradation rates constants, δ_i was obtained from differentiating mouse ES cells data (Sharova et al., *DNA Res*. **16**, 45–58 (2009)) and human B cells (Friedel et al., *Nucleic Acids Res.* **37**, e115 (2009)) and fitted to a lognormal distribution with mean (log scale) and standard deviation parameters, $\mu = -2.24$ and $\sigma = 0.61$ for mouse, and $\mu = -1.58$ and $\sigma = 0.73$ for human. (**B**) The time-averaged RNA transcription rates, T_i, were estimated from the same data, such as $T_i = x_i\delta_i$, where x_i is the steady state expression value of the ith gene, such as $\Pr(X = x_i) = x^{-0.8} / \sum_{k=1}^{20000} k^{-0.8}$. (Zipf's law with exponent $r = 0.8$, for a population of n = 20,000 genes. Exponent was estimated from the experimental data, see insert for a representative dataset (mouse and human blastocysts)). As a result, we fitted the estimated values of T_i to a lognormal distribution with parameters $\mu = 0.17$ and $\sigma = 2.67$ for mouse, and $\mu = 0.73$ and $\sigma = 2.53$ for human. To generate a random value for s_i, we first generate a value for T_i from the above lognormal distribution, and compute s_i, such as $s_i = T_i\,(k_{on,i} + k_{off,i})/(\phi_i k_{on,i})$, knowing ϕ_i, $k_{on,i}$ and $k_{off,i}$ beforehand.

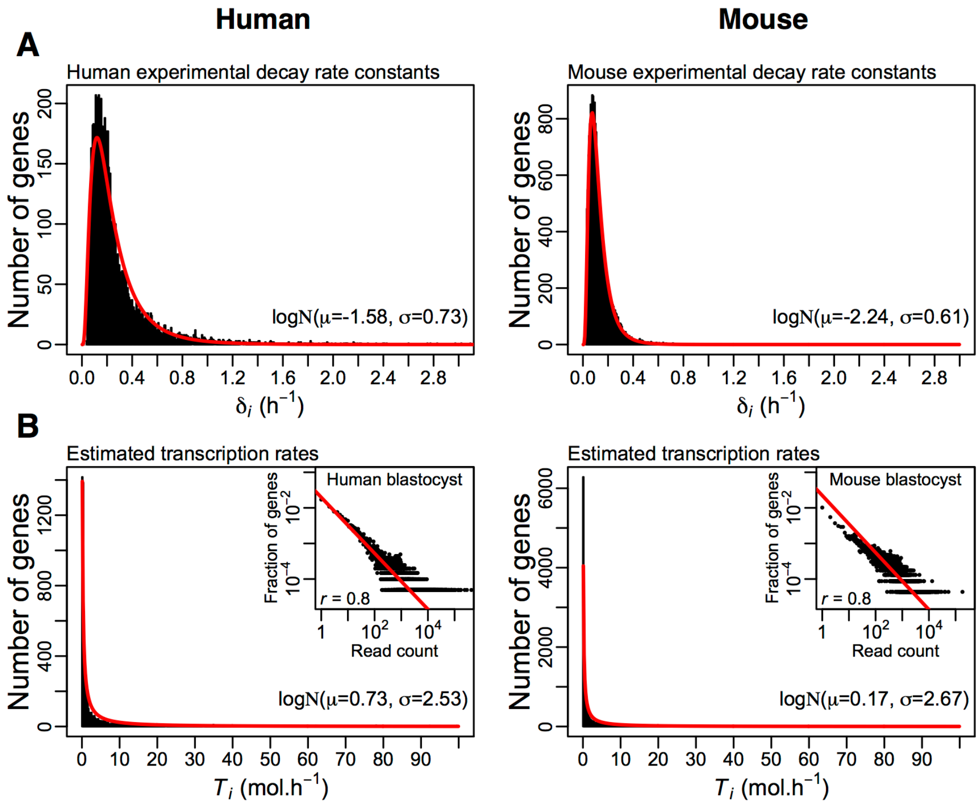

Figure S7. Simulated gene expression density distributions for each human and mouse development stage. Distributions were estimated using kernel density estimation (*kde2d* R function) of all genes expressions in the jth and k^{th} cell simulated transcriptomes for all pairs of single cells. See Figure 4C (maintext) for the parameters of the simulations. Blue and red colors indicate high density regions

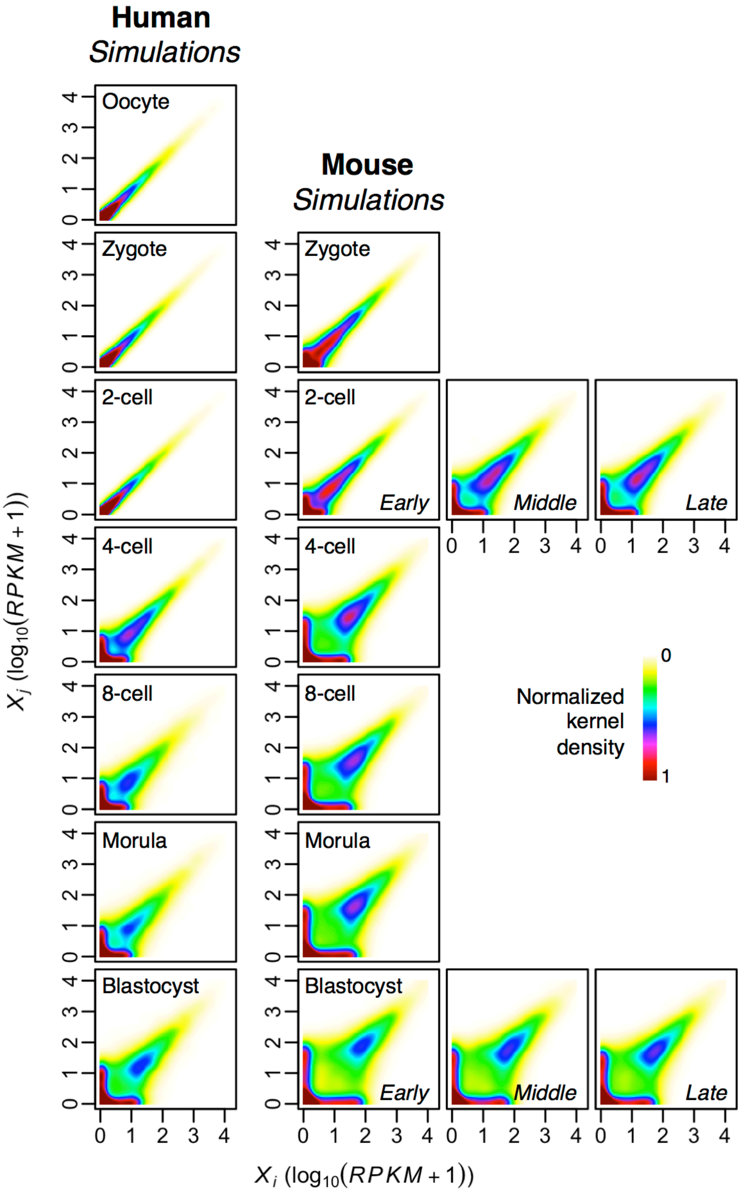

Figure S8. Fitting simulations to mouse experimental patterns. Panel 1: simulations (black curves) using continuous activation, no amplification and no extrinsic noise. Panel 2: adding extrinsic noise increases β. Panel 3: low transcriptional amplification increases α. This pattern fits early development stages (e.g. zygote, brown curve). Panel 4: higher amplification further increases α. Panel 5: switching from continuous to quantal activation further increases β. This pattern fits later development stages (e.g. early blastocyst, purple curve).

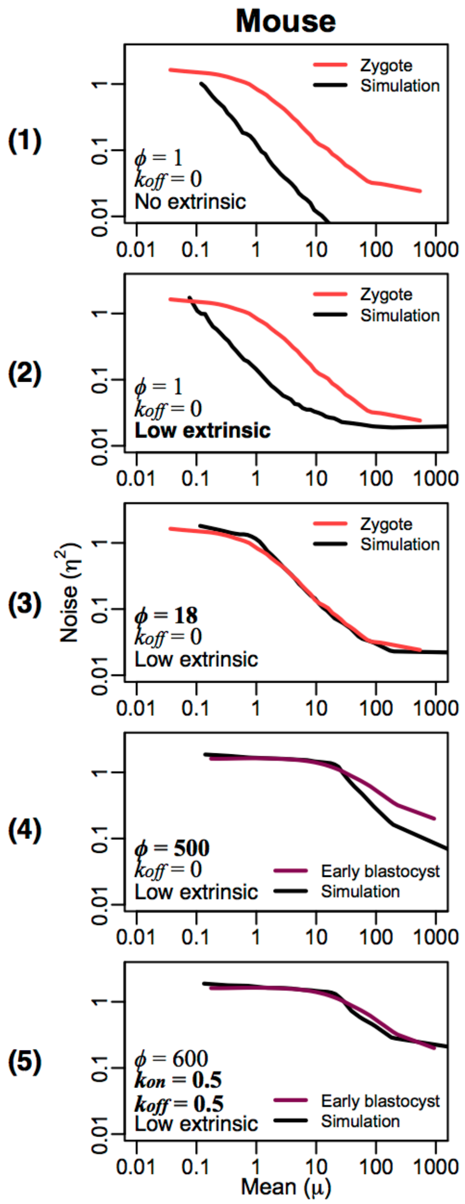

Figure S9. Gene expression profiles of maternal and zygotic genes during development. Individual expression profiles of **(A)** maternal (2 clusters) and **(B)** zygotic (3 clusters) genes during human embryo development. Average expression profiles of each cluster are shown in bold lines. Clusters were obtained using k-means clustering. From the initial lists of genes obtained from Xue et al., *Nature* **500**, 593–597 (2013) (see Table S1), we retained maternal genes with expression values reaching maximum before 4-cell stages and zygotic genes with expression values reaching maximum from 8-cell stage onwards.

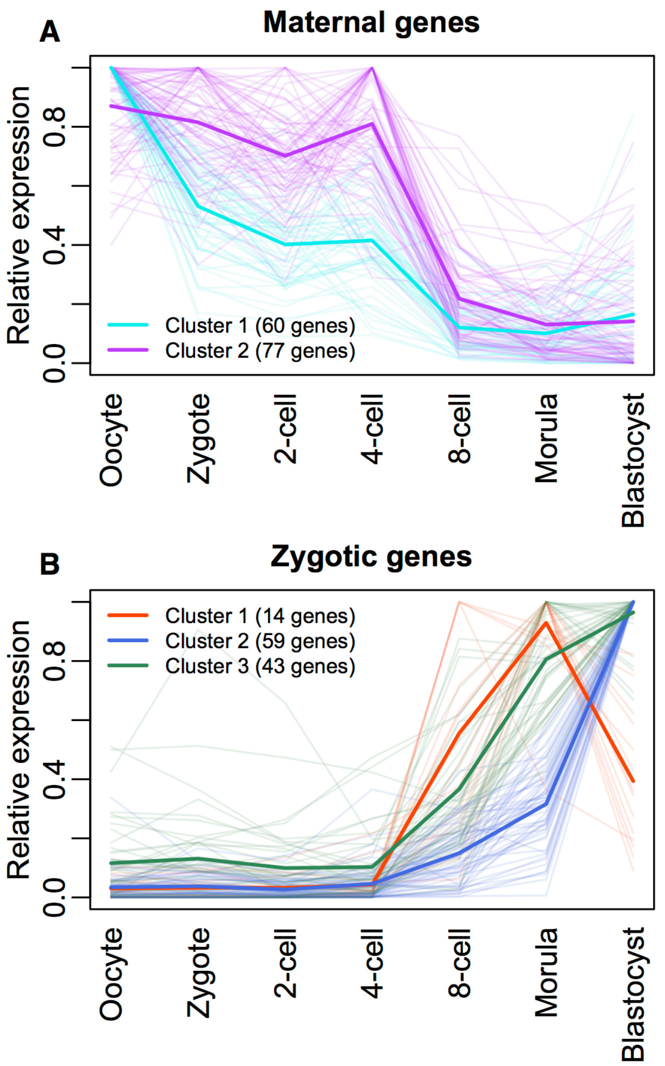

Table S1. List of genes activated at early stages (maternal) or late stages (zygotic)

Maternal genes (oocyte/zygote)			Zygotic genes (morula)		
Abca5	Grtp1	Skp1	Adck1	Klf8	Tmem33
Abcc4	H1foo	Skp2	Agl	Lama1	Tspan6
Abi3bp	Hook2	Snx25	Aimp1	Ldha	Tubb4
Acvr1b	Igsf11	Sp2	Akap12	Ldhb	Txn
Akd1	Ints9	Spire1	Arpc3	Lrpprc	Txndc12
Ampd3	Itgae	Stard7	Atp1b3	Lsm3	Uqcrb
Ankra2	Kif15	Stk38	Atp5f1	Lsm5	Uqcrh
Arhgef16	Klf11	Tapt1	Bcat1	Lypla1	Usmg5
Arv1	Klhdc3	Tdrd6	Btf3L4	Mbnl3	Wee1
Asf1b	Lamb1	Tfg	C14orf147	Mreg	Yipf4
Bcl2l10	Lancl1	Thoc7	C1orf109	Mrpl1	Zbtb8os
Blcap	Lmo3	Tifa	C2orf74	Mrpl27	
Bnip1	Lonrf1	Tmcc1	C4orf3	Mrps17	*Myc*[b]
C16orf57	Lrch1	Tmem163	C6orf211	Mrs2	*Sox2*[b,c]
C1orf210	Lrmp	Tnfsf13	C8orf59	Mtfmt	*Pou5f1*[b,c]
C1orf226	Lrrc16a	Trafd1	Cachd1	Mtif2	*Nanog*[c]
C22orf13	Lrrc49	Trak1	Cbx3	Ndc80	*Lin28a*[c]
C2orf29	Mal2	Troap	Ccdc72	Ndufb3	
C4orf27	Mctp2	Tspan7	Cct5	Olr1	
C5orf22	Meaf6	Tubg1	Cct6z	Pdcl2	
C5orf34	Mettl9	Tubg2	Cflar	Pet117	
C7orf10	Mfap3l	Ube2u	Chchd4	Pin4	
Casc3	Mknk1	Unkl	Cnih4	Ppap2a	
Cenpe	Mobkl1b	Wasf3	Cox7b2	Ppp2r5a	
Cep78	Mobkl3	Wdr69	Cpne3	Prim1	
Chic1	Mterfd1	Ypel1	Cpox	Prom1	
Ciapin1	Nars2	Zc3h7a	Csrp2Bp	Psma1	
Clec10a	Necap2	Zc3h8	Dapk1	Psmc6	
Clstn2	Nid2	Zfyve21	Denr	Ptplad1	
Cnn3	Nup133	Znf407	Dnajc25	Pum1	
Creb1	Nup37	Zswim3	Dsp	Rell1	
Dclk2	Pan2		Egflam	Rg9mtd2	
Dclre1a	Papd4	*Cdh3*[a]	Eif2s2	Rpl11	
Depdc7	Papd7	*Dppa5*[a]	F2r	Rpl17	
Dhx32	Papss1	*Mos*[a]	Fa2h	Rpl21	
Dmrtb1	Pcmtd1	*Npm2*[a]	Fam108c1	Rpl22	
Dnajc3	Per3	*Zp1*[a]	Fbxo22	Rpl23	
Dpf2	Polb	*Zp2*[a]	Gde1	Rpl34	
Dusp19	Ppil2		Gdpd1	Rpl39L	
Dusp7	Ptprk		Gja1	Rpl7	
Dync2h1	Rai14		Glud1	Rps27a	
Epb41l5	Rapgef6		Guf1	Rrm1	
Fbxo43	Rexo2		Gulp1	S100a11	
Fdft1	Rfesd		Havcr1	Sdhd	
Ftl	Rhot1		Hddc2	Serbp1	
Ggct	Rnf10		Hpgd	Shfm1	
Gnpda2	Rpia		Ibtk	Skiv2l2	
Gopc	Sdccag8		Ipo5	Slc35b3	
Grb7	She		Klf3	Slco4c1	
Grm8	Shmt2		*Klf4*[b]	Ssbp1	

Note: to the initial lists of genes obtained from Xue et al. 2013, we added maternal genes from **(a)** Kocabas et al., *Proc. Natl. Acad. Sci. U. S. A.* **103**, 14027–14032 (2006), and genes expressed in stem cells from **(b)** Takahashi et al., *Cell* **131**, 861–872 (2007) and **(c)** Yu et al., *Science* **318**, 1917–1920 (2007).
